# Puncture Resistance and UV aging of Nanoparticle-Loaded Waterborne Polyurethane-Coated Polyester Textiles

**DOI:** 10.3390/ma16216844

**Published:** 2023-10-25

**Authors:** Domenico Acierno, Lucia Graziosi, Antonella Patti

**Affiliations:** 1 Regional Center of Competence New Technologies for Productive Activities Scarl, Via Nuova Agnano 11, 80125 Naples, Italy; lucia.graziosi93@gmail.com; 2 Department of Civil Engineering and Architecture (DICAr), University of Catania, Viale Andrea Doria 6, 95125 Catania, Italy; antonella.patti@unict.it

**Keywords:** nanoparticles, waterborne polyurethane, UV aging, mechanical resistance, polyester fabrics

## Abstract

The goal of this research was to investigate the effect of different types of nanoparticles on the UV weathering resistance of polyurethane (PU) treatment in polyester-based fabrics. In this regard, zinc oxide nanoparticles (ZnO), hydrophilic silica nanoparticles (SiO_2_ (200)), hydrophobic silica nanoparticles (SiO_2_ (R812)), and carbon nanotubes (CNT) were mixed into a waterborne polyurethane dispersion and impregnated into textile samples. The puncturing resistance of the developed specimens was examined before and after UV-accelerated aging. The changes in chemical structure and surface appearance in nanoparticle-containing systems and after UV treatments were documented using microscopic pictures and infrared spectroscopy (in attenuated total reflectance mode). Polyurethane impregnation significantly enhanced the puncturing strength of the neat fabric and reduced the textile’s ability to be deformed. However, after UV aging, mechanical performance was reduced both in the neat and PU-impregnated specimens. After UV treatment, the average puncture strength of all nanoparticle-containing systems was always greater than that of aged fabrics impregnated with PU alone. In all cases, infrared spectroscopy revealed some slight differences in the absorbance intensity of characteristic peaks for polyurethane polymer in specimens before and after UV rays, which could be related to probable degradation effects.

## 1. Introduction

In 2019, synthetic fibers accounted for more than 60% of global consumption of natural and man-made fibers, with polyester accounting for 53%, cotton accounting for 24%, cellulosic accounting for 6%, and wool accounting for 1%.

Polyester fibers have a specific gravity of 1.22–1.38 g/cm^3^ [1], are wrinkle resistant [2], and have a moisture regain of roughly 0.4%. Mechanical properties are frequently dependent on fiber drawing; as the polymer chain alignment increases, so does the tensile strength and modulus of polyester fibers [3]. PET fibers are widely used in woven and knitted garments and upholstery, including pants, skirts, stockings, carpets, and blankets, as well as industrial threads and ropes, protective textiles (work wear, conveyor belt), and jet engines (abradable seal) [3].

Coating or impregnation are two common methods, generally considered in the textile industry, for improving the final performances of treated fabrics and providing a proper finishing [4]. In recent years, many researchers investigated the puncture resistance behavior of textile materials, and it was discovered that the perforating properties of textiles were affected by coating technologies [5]. The body protection for law enforcement, security, and military personnel has been the primary focus of investigations on the stab and puncture resistance of fabrics [6]. Firouzi et al. investigated nylon-based coating to improve the penetration resistance of ultra-high molecular weight polyethylene (UHMWPE) against spikes or blades [7]. The puncture performance of woven high modulus polypropylene (HMPP) fabric impregnated with dispersions constituted of fumed silica nanoparticles, carbon nanotubes (CNTs), and polyethylene glycol (PEG) was investigated in [8].

Polyurethane is widely employed in advanced coating technology because it improves the quality, appearance, and durability of coated substrates [9]. A polyurethane and pre-vulcanized natural rubber latex coating was tested on polyester textiles to obtain high performance in water vapor permeability, water pressure resistance, and stretchability [10]. Polyurethane formulations have traditionally been manufactured with hazardous organic solvents such as toluene, xylene, and formaldehyde. Its manufacturing process is responsible for volatile organic compounds emissions, relative ozone formation potential, and relative carcinogenic risk [11,12]. However, green materials have become popular in the coating industry as a result of growing environmental concerns and the depletion of petroleum supplies [13]. As an alternative to solvent-based polyurethane dispersions, water-based polyurethane dispersions (WPU) with minimal volatile organic compounds (VOCs) and no co-solvents were developed [14]. The addition of nanoparticles to WPU has been demonstrated useful to improve the overall performance of WPU-based products and attain the same requirements as their solvent-based equivalents [11,15].

Recently, nanomaterials have been used to modify the characteristics of organic and inorganic matrices and improve their durability against UV weathering. Polyetheretherketone (PEEK) was loaded with sub-micrometric titanium dioxide particles to increase the UV resistance of the basic polymer [16]. Gorrasi and Sorrentino examined the influence of carbon nanotubes on the photo-oxidation of composites made of polylactic acid [17]. Cheraghian and Wistuba investigated the effect of fumed silica nanoparticles on the mechanical and chemical properties of bitumen after UV aging [18]. The addition of nano-zinc oxide particles was discovered to minimize the photo-degradation of the polyurethane film and shield it from the detrimental impacts of UV light [19].

In this investigation, various types of nanoparticles (zinc oxide nanoparticles, hydrophilic and hydrophobic silica nanoparticles, and carbon nanotubes) were combined into aqueous polyurethane dispersions and impregnated into polyester-based fabrics. The mechanical performance of produced specimens was evaluated mainly in terms of puncture strength. The measurement of fabric puncture strength has been regarded as a practical and useful approach to analyzing the quality of textiles used in daily life, particularly in the industry production of bags, luggage, and suitcases. Puncture resistance might represent a frequent stress condition to which materials may be subjected during common uses, potentially damaging and compromising integrity and functionality. To verify treatment durability, mechanical properties were also evaluated following an accelerated aging process. Environmental variables such as UV radiation, heat from the sun, and moisture can cause polymer-based materials to degrade by affecting mechanical properties and limiting product life span. Understanding the effects of UV radiation on the mechanical performance of textiles can be useful in predicting the durability of final products. Infrared spectroscopy and images of sample surfaces were collected on specimens before and after UV exposure as primary characterization tools to analyze the chemical and visible changes caused by potential breakdown of the applied PU polymer.

## 2. Materials and Methods

### 2.1. Materials

In this study, a technical fabric (FAB) with a woven structure constructed entirely of polyester fibers (100%) and with an area density of roughly 300 g/m^2^ was explored. The aliphatic polyester-based polyurethane waterborne dispersion (IDROCAP 983 PF, anionic nature, dry residue at 130 °C 1 h = 35%, pH = 7.8) was kindly supplied by ICAP-Sira Chemicals and Polymers s.p.a., Parabiago-Milan, Italy. Hydrophilic fumed nano-silica (here identified as SiO_2_ (200)) (cod. AEROSIL 200, an average particle size of 12 nm, a specific surface area of 175–225 m^2^/g) and hydrophobic organosilane modified silica produced by treating fumed SiO_2_ with hexamethyldisilazane (HMDS) (here identified as SiO_2_ (R812)) (cod. AEROSIL R812, an average particle size of 7 nm, a specific surface area of 260 ± 30 m^2^/g) were acquired by Evonik Resource Efficiency GmbH (Germany). Carbon nanotubes (CNT) (cod. NC 3150, length < 1 μm, diameter: 9.5 nm and purity > 95%) were supplied by Nanocyl S.A. (Sambreville—Belgium). Zinc oxide (ZnO, average particle size of 100 nm) was purchased from Sigma Aldrich Co. LLC., Milan, Italy.

### 2.2. Sample Preparation

A square piece of fabric (20 cm in size) was soaked in 13 g of aqueous polyurethane (Fab-PU). The wet specimens were dried in a climatic chamber (mod. 250E, Angelantoni Industrie spa, Perugia, Italy) at 25 °C and 50% humidity. Other impregnating dispersions were produced by combining the components, namely nanoparticles (hydrophilic and hydrophobic SiO_2_, ZnO, CNT) and aqueous polyurethane (13 g), for 15 min with magnetic stirring at 800 rpm. The nanoparticle weight contents of 1% and 4% were related to the nominal solid polyurethane component in the dispersion (see details in Table 1). A dry time of about 4 days was determined through preliminary testing when the sample weight stabilized with time.

UV-accelerated aging was carried out for 5 days at 35 °C in an oven equipped with a low-pressure mercury lamp (90% irradiation at 254 nm, 10% irradiation at 185 nm).

### 2.3. Characterization Techniques

**Figure 1:**
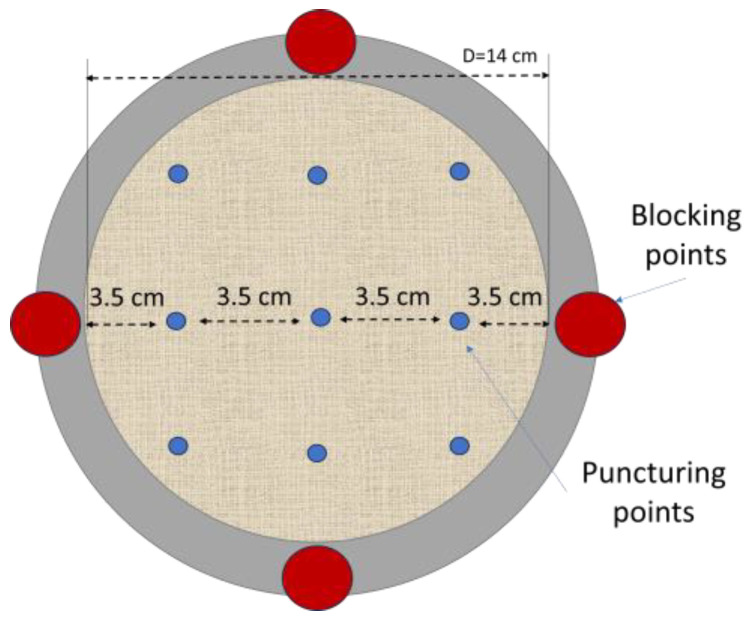
Schematic of puncturing positions on the sample surface.

The puncture resistance of the treated fabrics was evaluated on a dynamometer (cod. TENSOMETER 2020) produced by Alpha Technologies INSTRON (Norwood, MA, USA). The specimens were held between two annular flanges with internal diameters of 14 cm, and firmly secured with four screws. The upper mobile grip was equipped with a rounded tip spike (3 mm in diameter) and traveled down perpendicular to the sample at a speed of 50 mm/min. Tensile 2020 was the management software. The maximum force sustained by the specimen before breaking corresponds to the highest point achieved on the load–displacement curve. This value was considered the puncture resistance. Measurements were repeated 9 times for each sample. Two different specimens were prepared and tested for each formulation. The maximum change in material length before breaking caused by the piercing probe’s perpendicular pulling force was identified as displacement. Figure 1 depicts the locations of the punching points on the sample surface during testing.

An infrared spectrometer (model Spectrum 65 FT IR) from Perkin Elmer (Waltham, MA, USA) was used to examine aged specimens in attenuated total reflectance (ATR) mode. During the test, infrared radiation was transmitted via a diamond crystal upon which the sample was placed. Initially, the background spectrum was collected using an empty and clean crystal. A wavenumber range of 650–4000 cm^−1^, a resolution of 4 cm^−1^, and a scan number of 16 were set during the experiment.

The effect of impregnating dispersions on the appearance of the textile surface was examined through the auxilium of a Wi-Fi digital microscope 1000× with manual focal length (resolution 1280 × 1024).

## 3. Results

### 3.1. Visual Aspects of Sample Surface

**Figure 2:**
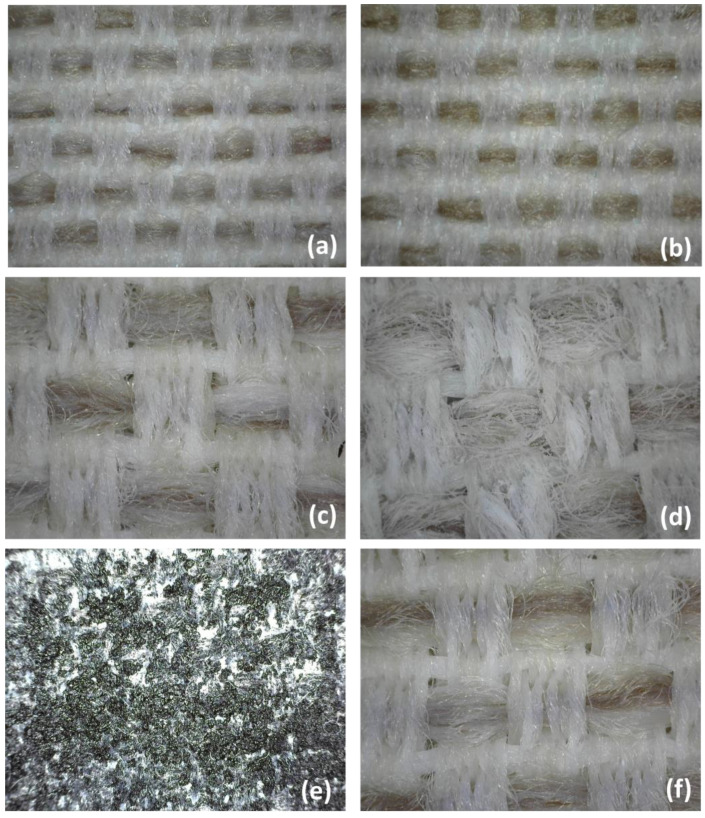
Images of sample surfaces for (**a**) FAB; (**b**) FAB-PU; (**c**) FAB-PU + 4% SiO_2_ (200); (**d**) FAB-PU + 4% SiO_2_ (R812); (**e**) FAB-PU + 4% CNT; (**f**) FAB-PU + 4% ZnO.

The effect of the treatment on the final aesthetics of the fabric was evaluated in relation to the color of the base surface. In this instance, a beige/white fabric was used. The surface appearance of the textile surface of each prepared specimen was reported in Figure 2.

The textile impregnation with aqueous polyurethane (Figure 2b) did not appear to significantly alter the surface properties of basic fabric (Figure 2a). A very thin layer seemed to coat the fibers on the surface, making them slightly brighter.

When the hydrophilic silica and zinc oxide nanoparticles were added into dispersion (Figure 2c,f, respectively), only at the microscopic level an aesthetic change in the visible characteristics of the material can be discerned, becoming less brilliant and whiter. The presence of nanoparticles in the soaking solution had a significant impact when the hydrophobic nanosilica or carbon nanotubes were embodied in the aqueous medium, as seen in Figure 2d,e, respectively. In the first situation, the fabric surface was colored with a brighter white, whereas in the second case, the fabric surface darkened.

Touching and stroking the treated fabric sample resulted in no nanoparticle detachment or dispersion in the surroundings. Polyurethane was found to be necessary in order to adhere the nanoparticles to the textile structure and prevent their leakage into the environment during use.

**Figure 3:**
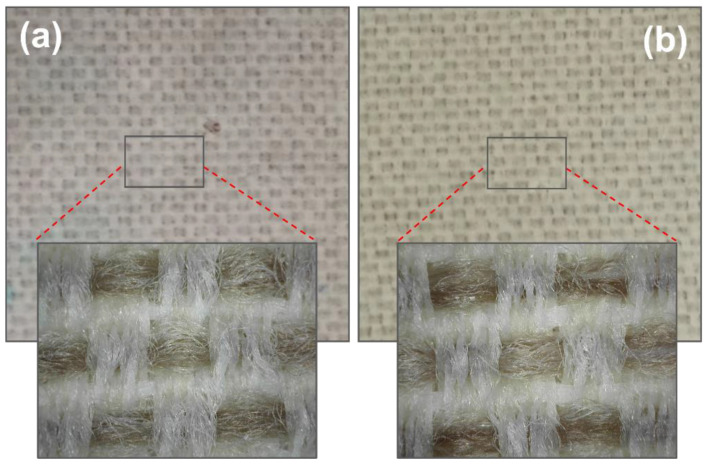
Impregnated textiles with WPUD containing 4% in wt. of hydrophilic nano-SiO_2_ before (**a**) and after (**b**) UV treatment.

Although the effectiveness of keeping the physical and mechanical properties of the polymer is critical, there is frequently another issue that can cause concerns regarding the product quality: “Yellowing or Pinking”. This color-change issue could severely limit the final product approval. Such discoloration was mainly attributed to a side reaction of the polymer itself during the manufacturing process or to by-products of the stabilization processes of antioxidants and stabilizers [20]. By comparing the color of aged samples to the initial ones at the end of the UV irradiation time, a yellowing phenomenon of the textile surface in specimens incorporating all the different types of nanoparticles (ZnO and hydrophilic and hydrophobic silica) could be observed (Figure 3).

Thus, the presence of particles appeared ineffective in preventing the yellowing of treated textiles. Due to the darker surface in CNT-based specimens, this effect was not evident.

Typically, several research findings highlighted the benefits of incorporating nanoparticles into a polymer matrix to minimize UV-induced photo-oxidation, which was thought to be the primary cause of polymer yellowing. For example, micrometric zinc oxide particles were found to improve the stability against UV and hydrophobicity without affecting the final mechanical performance of polyester-coated specimens [21]. The optimal ZnO content for impregnated fabrics was set at 3–5 wt.%. The photoprotective effect was attested through UV reflectance measurements via a spectrometer [21]. Zinc oxide nanoparticles were synthesized and applied on cotton and wool fabrics for UV shielding in [22]. Assessments of the treatment’s efficiency were based on UV-Vis spectrophotometry and the computation of the ultraviolet protection factor (UPF). Grigoriadou et al. [23] studied the UV stability of high-density polyethylene (HDPE) with several types of nanoparticles (montmorillonite, silica, and carbon nanotubes). Nanocomposites were aged using UV irradiation at 280 nm at a constant temperature (25 °C) for up to 200 h. The results showed a first increase in tensile characteristics after 100 h of irradiation, followed by a decrease, with an effect more evident in the presence of nanoparticles. FTIR measurements revealed a significant degradation of HDPE polymer, especially with the addition of silica and montmorillonite. The authors concluded that such particles enhanced HDPE photo-oxidation.

### 3.2. Puncture Performance

**Figure 4:**
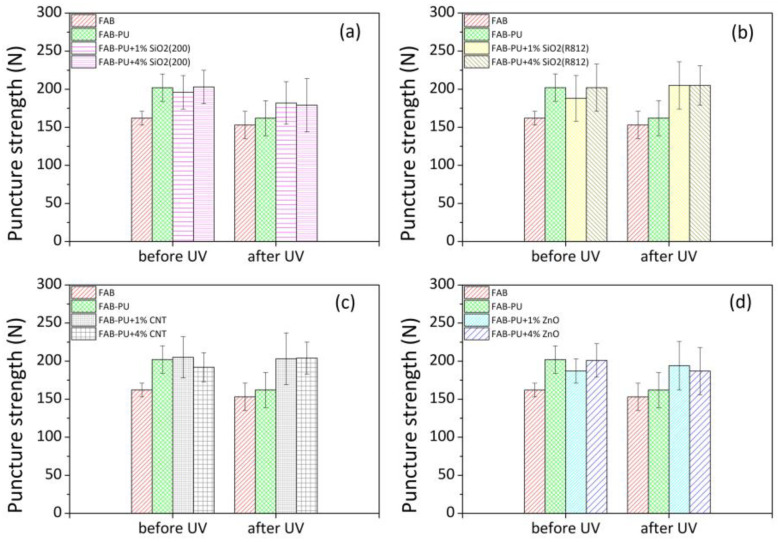
Average puncture strength of PU-impregnated specimens containing (**a**) hydrophilic SiO_2_; (**b**) hydrophobic SiO_2_; (**c**) CNT; (**d**) ZnO before and after UV aging.

Table 2 summarizes the puncture load and displacement for impregnated fabrics containing zinc oxide, hydrophilic silica, hydrophobic silica, and carbon nanotubes before and after UV aging. For comparison, data from basic fabric and impregnated fabric have been reported. Results were displayed in terms of mean (MN), median (MD), maximum (MAX) and minimum (MIN) values, and standard deviations (STD). Figure 4 summarizes the average puncture load for each developed system before and after UV.

**Figure 5:**
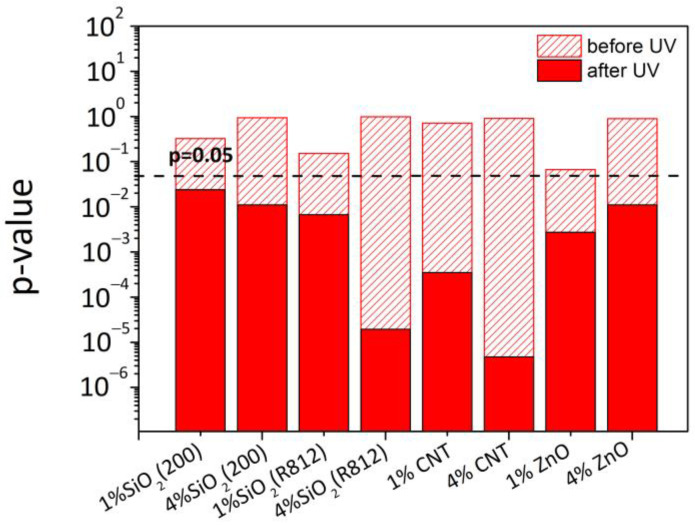
*p*-values from the analysis of variance of the puncture strength of PUD-impregnated specimens in comparison to FAB-PU systems before and after UV exposure.

There were very small effects of UV radiation on the mechanical performance of the basic material (FAB specimens). The original polyester had an average puncturing load of 162 N, which was 153 N after UV treatment. On the contrary, a strong decrease in puncture strength was seen for impregnated textiles (FAB-PU samples). In this case, the average load started at ~202 N and arrived at ~162 N. The negative impact of UV weathering on the benefits of polyurethane treatment of the fabric surface was attributed to the potential destruction of molecular bonds in the PU polymer applied to the textile surfaces (as further confirmed through ATR measurements). The presence of nanoparticles in polyurethane dispersion, regardless of filler type, did not significantly increase mechanical resistance against the piercing probe. However, the reinforcing effect of nanoparticles was observed following UV aging. The puncturing strength of aged specimens containing nanoparticles was superior to that of aged specimens treated only with PU. The data was statistically examined with analysis of variance at one factor (One-way ANOVA) comparing values from treated samples with polyurethane loaded with nanoparticles to ones from samples treated solely with polyurethane before and after UV (Figure 5). *p*-value approach was used to interpret the relevance of differences. When p is less than 0.05 (*p* < 0.05), there is enough evidence to determine that the effect is significant.

Initially (before UV), systems containing nanoparticles behave similarly to systems impregnated with polyurethane alone (*p* > 0.05). After UV, in all cases, *p*-values were much lower than 0.05. This underlined the strong effect of nanoparticles on the puncture strength of treated textiles, particularly in the case of carbon nanotubes and hydrophobic silica nanoparticles.

The average displacement of the beginning fabric, which was roughly 28 mm, was slightly reduced by treating the textile with the aqueous polymer solution. When nanoparticles were added to the impregnating dispersion, especially carbon nanotubes, the capacity of the textile to be deformed during the puncturing test was reduced even further by stiffening the textile structure. After UV exposure, the treated textiles became more and more rigid and less deformable.

These findings were consistent with previous literature studies on the same topics. Polyurethane served as a protective support in basic textiles, preserving and strengthening the fibers and threads. Common coating and laminating processes were demonstrated to be effective in enhancing the stiffness of treated fabrics because the applied polymer on textile structures operated by binding together weft and warp threads. Mechanical strength increased as a result of more filaments sharing the mechanical load. The previous research activities demonstrated a variety of advantages to using polyurethane in the textile weave of synthetic fabrics [24,25,26]. The impregnation treatment had no influence on the appearance of virgin textile material despite an increase in rigidity and weight. PU-treated samples outperformed untreated samples in terms of breaking tensile load, abrasion resistance, water repellency, and waterproofness. [25]. The effect of aqueous polyurethane dispersion containing hydrophilic or hydrophobic silica nanoparticles on the quasi-static perforating features of polypropylene-based textiles was attested in [24]. As piercing probes, a spherical spike and a pointed blade were used in quasi-static perforation experiments. The most significant results, i.e., the greatest rise in blade strength and piercing strength compared to the neat material, were obtained through the incorporation of the two additives, nano SiO_2_ and crosslinker, into the polyurethane. The beneficial effect of nano SiO_2_ on the mechanical properties of produced textiles was attributed to the efficient inhibition of fibers from sliding easily during the puncture testing by determining more energy spent in friction.

### 3.3. Infrared Spectroscopic Measurements

FT-IR spectroscopy, in attenuated total reflectance mode, was utilized to identify and qualitatively analyze the chemical changes caused by ultraviolet irradiation in polyurethane-based systems.

**Figure 6:**
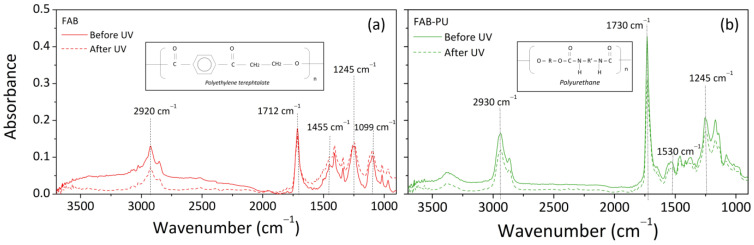
ATR spectra before and after UV of FAB (**a**) and FAB-PU (**b**) specimens.

The ATR-FTIR spectra of basic and treated polyester-based fabrics (before and after UV weathering) were analyzed in Figure 6.

From the left to the right, different absorption bands were recognized for the basic PET textile [27] (Figure 6a): (i) at 2920 cm^−1^ and 2850 cm^−1^ assigned to asymmetrical and symmetrical stretching of methylene groups (CH_2_); (ii) at 1712 cm^−1^ due to the stretching of carboxylic ester group (C=O); (iii) at 1455 cm^−1^ assigned to C–H deformation; (iv) at 1245 cm^−1^ caused by asymmetric C-C-O stretching in the aromatic ring; (v) at 1095 cm^−1^ due to the ester C–O–C stretching. The absorption peak at 1407 cm^−1^, corresponding to the aromatic ring, was the characteristic peak of PET [28].

A board band at 3450 cm^−1^ was found in basic textiles before UV aging, usually associated with the stretching of hydroxyl groups. In this case, this band could be due to the possible moisture absorption on the sample surface, potentially removed during the UV treatment in the oven at 35 °C. Furthermore, the polyester structure includes ester, alcohol, anhydride, aromatic rings, and heterocyclic aromatic rings. Alcohol could react with anhydride to form ester groups. This indicates that the polyester could still contain residual reactants such as alcohol and anhydride [28]. The peaks at 1712 and 1095 cm^−1^ were considered a sign of polyester breaking under certain conditions [28]. Except for the region between 3500 and 2500 cm^−1^, there was no significant alteration in the base textiles downstream of the UV aging compared to the original scenario.

On the other side, the main characteristic bands of polyurethane polymer were distinguished in treated polyester-based textiles (Figure 6b): N-H stretching vibrations in the range of 3500–3300 cm^−1^, N-H bending and C-N stretching at 1530 cm^−1^, C-H asymmetric and symmetric stretching at 2930 cm^−1^ and 2855 cm^−1^, respectively, C=O stretching at 1730 cm^−1^, and C-O vibration at 1245 cm^−1^ [29,30]. The distinctions in spectra of basic PET and samples treated with PU confirmed the efficiency of the treatment on the sample’s surface.

**Figure 7:**
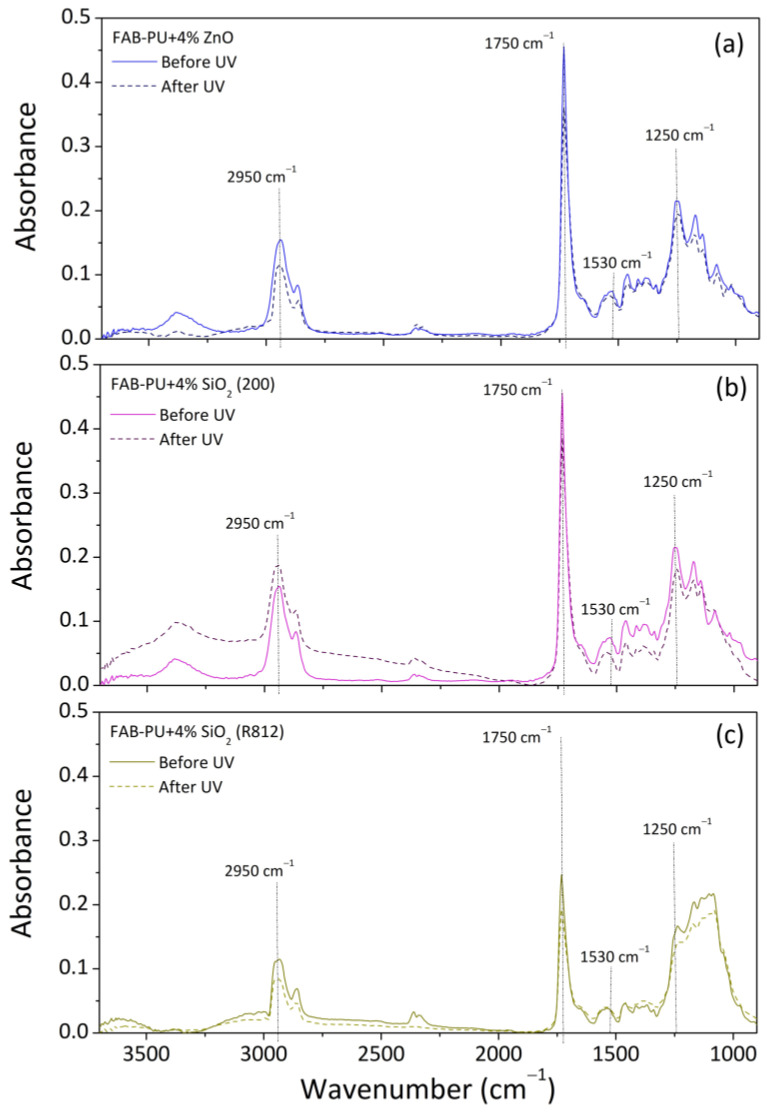
ATR spectra before and after UV of (**a**) FAB-PU + 4% ZnO; (**b**) FAB-PU + 4% SiO_2_ (200); (**c**) FAB-PU + 4% SiO_2_ (R812).

A comparison of ATR spectra of samples before and after UV aging was reported in Figure 7 for systems containing zinc oxide (a), hydrophilic silica (b), and hydrophobic silica (c). This investigation was not performed in the case of systems containing carbon nanotubes since this filler is a black substance that absorbed all of the radiation, resulting in a signal with a lot of noise. Li et al. [31] reported infrared spectroscopic measurements on basic, ammino-treated, or acid-treated multi-walled carbon nanotubes. In the instance of unfunctionalized carbon nanotubes, the signal was just noisy, with no evidence of specific peaks or absorption bands.

In all the cases, the peak intensity of the aforesaid bands (typical of PU polymers), intended as the absorbance value at the maximum point, was slightly decreased in specimens after UV exposure. This result was considered a sign of polyurethane chemical degradation following the UV treatment. The loss in absorbance (L_λ_) was calculated as the ratio of the recorded intensities at the specific wavenumbers (λ) of 2950 cm^−1^, 1750 cm^−1^, 1530 cm^−1^, and 1250 cm^−1^ before UV ${(A}_{beforeUV}^{\lambda })$ and after UV ${(A}_{afterUV}^{\lambda })$ (Table 3) according to Equation (1):

(1) $$L_{\lambda }:\frac{A_{afterUV}^{\lambda }}{A_{beforeUV}^{\lambda }}$$

**Figure 8:**
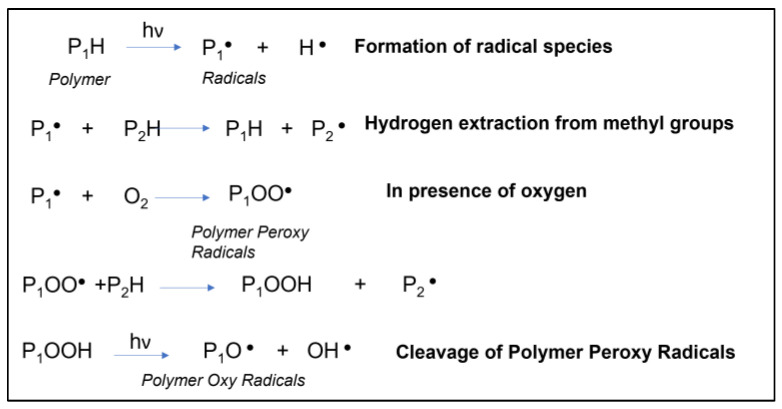
Possible reactions occurred during the UV-induced degradation of PU polymer.

Depending on whether the PU polymer is aliphatic or aromatic, photo-oxidation could take different courses. In the case of aliphatic PU (as in this case study), firstly, a mechanism of random chain scission could occur (Norrish Type I reaction) by leading to the formation of free radicals (Figure 8). These radical species could abstract hydrogen from CH_2_ groups and generate polymer peroxy radical (PO_2_^•^) and polymer hydroperoxide (POOH). The POOH could then be converted into polymer oxy (PO^•^) radicals if the scission reaction occurs, or hydroxyl group (POH) if the abstraction hydrogen reaction occurs. The degradation pathway could end with the interaction of different radicals with each other via the crosslinking reaction [32].

As concerning the nanoparticles, zinc oxide displayed an absorption signal at wavenumbers less than 500 cm^−1^, not reached in the present case [33]. Hydrophilic silica (Aerosil 200) displayed two characteristic peaks in the range of 1050–780 cm^−1^ attributed to the asymmetric–symmetric vibration of the Si-O-Si bond (siloxane groups) [34]. Hydrophobic silica (Aerosil R812) displayed a recognizable peak in areas of C-H stretching bands (2850–3000 cm^−1^), considered correlated to the alkyl groups of organo-silane modifiers on the surface of silica nanoparticles, and the main peak at 1100 cm^−1^ corresponding to the symmetric Si-O-Si stretching [35].

Following UV irradiation, the intensity of absorption bands of hydroxyl radicals (OH•) in the area of 3600–3200 cm^−1^ rose only for FAB-PU + 4% SiO_2_ specimens. This could be due to the formation of hydroxyl radicals during the cleavage of polymer peroxy radicals (as indicated in the last reaction reported in Figure 8), which were next linked to silica, given the hydrophilic nature of nanoparticles.

## 4. Conclusions

This work investigated the mechanical characteristics, in terms of puncture strength, of impregnated polyester-based textiles with aqueous polyurethane solutions containing different types of nanoparticles, such as zinc oxide, functionalized silica (both hydrophilic and hydrophobic), and carbon nanotubes. All systems were evaluated using a puncturing test, infrared spectroscopy, and microscopic examination before and after UV-accelerated aging.

The results revealed a change in the appearance of treated specimens containing nanoparticles, particularly hydrophobic silica and carbon nanotubes. Such nanoparticles remained more distributed on the sample surface without spreading onto the textile structure. Furthermore, in the case of CNT, the textile surface retained its black hue. The mechanical resistance of PU-impregnated specimens was raised compared to basic fabric. A negligible effect on the average puncture strength of PU-treated textiles was verified when nanoparticles were introduced to the textile treatment. On the other side, the stiffness of neat fabric became higher when the polyurethane was applied to the textile surface.

Following UV aging, the yellowing effect was also visible in nanoparticle-containing specimens. A loss of mechanical performance was verified for basic fabric and PU-impregnated specimens. When nanoparticles were present on the fabric surface, the average puncture strength of treated specimens remained greater than the average value obtained in fabric treated with polyurethane alone (without nanoparticles). This was attributed to an increase in energy spent in friction during the passage of the puncturing probe in the textile surface covered by nanoparticles. In such systems, infrared spectroscopy revealed a weak drop in absorbance intensity for polyurethane absorption bands, intended as potential polymer deterioration during UV weathering.

Finally, the addition of nanoparticles to impregnated fabrics did not significantly inhibit polymer deterioration after UV treatment, as evidenced by yellowing in treated specimens. However, the nanoparticles seemed to positively contribute as reinforcement of PU polymer into the textile structure to overall improve the mechanical strength, especially after UV exposure.

## Figures and Tables

**Table 1:** Developed formulations to impregnate a single piece of fabric (20 × 20 cm^2^).

	WPU	ZnO	SiO_2_ (200)	SiO_2_ (R812)	CNT
FAB-PU	13 g	/	/	/	/
FAB-PU + 1% ZnO	13 g	1% in wt. (* = 0.04 g)	/	/	/
FAB-PU + 4% ZnO	13 g	4% in wt. (* = 0.18 g)	/	/	/
FAB-PU + 1% SiO_2_ (200)	13 g	/	1% in wt. (* = 0.04 g)	/	/
FAB-PU + 4% SiO_2_ (200)	13 g	/	4% in wt. (* = 0.18 g)	/	/
FAB-PU + 1% SiO_2_ (R812)	13 g	/	/	1% in wt. (* = 0.04 g)	/
FAB-PU + 4% SiO_2_ (R812)	13 g	/	/	4% in wt. (* = 0.18 g)	/
FAB-PU + 1% CNT	13 g	/	/	/	1% in wt. (* = 0.04 g)
FAB-PU + 4% CNT	13 g	/	/	/	4% in wt. (* = 0.18 g)

(*) with respect to PU solid content in waterborne polyurethane dispersion (4.55 g of solid PU).

**Table 2:** Statistical analysis of puncture data (load and displacement) for PU-impregnated specimens: mean (MN), median (MD), maximum (MAX) and minimum (MIN) values, and standard deviation (STD).

	Load					Displacement				
Before UV	MN	MD	MIN	MAX	STD	MN	MD	MIN	MAX	STD
FAB	162	157	152	182	9	28	26	21	51	7
FAB-PU	202	201	170	244	28	26	26	15	35	20
FAB-PU + 1% ZnO	187	188	158	214	16	20	18	15	26	4
FAB-PU + 4% ZnO	201	197	161	241	22	21	21	14	35	5
FAB-PU + 1% SiO_2_ (200)	196	195	153	239	22	22	21	12	33	6
FAB-PU + 4% SiO_2_ (200)	203	205	153	237	22	21	19	13	33	6
FAB-PU + 1% SiO_2_ (R812)	188	190	127	246	30	19	17	11	44	8
FAB-PU + 4% SiO_2_ (R812)	202	211	162	250	31	19	18	12	27	5
FAB-PU + 1% CNT	205	207	161	244	27	18	18	12	27	3
FAB-PU + 4% CNT	203	204	144	240	34	19	19	15	33	4
**After UV**										
FAB	153	149	134	194	18	25	23	16	44	7
FAB-PU	162	156	134	201	23	20	18	15	33	5
FAB-PU + 1% ZnO	194	197	144	261	32	20	19	14	31	5
FAB-PU + 4% ZnO	187	181	139	258	31	19	18	13	29	4
FAB-PU + 1% SiO_2_ (200)	182	184	137	246	28	18	17	10	29	5
FAB-PU + 4% SiO_2_ (200)	179	180	127	237	35	19	17	10	38	7
FAB-PU + 1% SiO_2_ (R812)	189	190	130	239	31	18	17	13	31	5
FAB-PU + 4% SiO_2_ (R812)	205	205	163	245	26	17	16	11	27	4
FAB-PU + 1% CNT	192	200	159	212	19	16	17	11	23	3
FAB-PU + 4% CNT	204	205	162	235	21	16	15	12	23	4

**Table 3:** Loss in absorbance ($L_{\lambda }:$ L_2930,_ L_1730,_ L_1530,_ L_1245_) at the respective wavenumber (λ: 2950 cm^−1^, 1750 cm^−1^, 1530 cm^−1^, 1250 cm^−1^) for each specimen.

	L_2930_	L_1730_	L_1530_	L_1245_
FAB-PU	0.73	0.72	0.80	0.84
FAB-PU + 4% ZnO	0.75	0.85	0.67	0.81
FAB-PU + 4% SiO_2_ (200)	1.25	0.85	0.66	0.80
FAB-PU + 4% SiO_2_ (R812)	0.77	0.83	0.89	0.87

## Data Availability

The data presented in this study are available on request from the corresponding author.
